# Development of a Novel Sphingolipid Signaling Pathway-Related Risk Assessment Model to Predict Prognosis in Kidney Renal Clear Cell Carcinoma

**DOI:** 10.3389/fcell.2022.881490

**Published:** 2022-06-29

**Authors:** Yonghao Sun, Yingkun Xu, Xiangyu Che, Guangzhen Wu

**Affiliations:** ^1^ Department of Urology, The First Affiliated Hospital of Dalian Medical University, Dalian, China; ^2^ Department of Endocrine and Breast Surgery, The First Affiliated Hospital of Chongqing Medical University, Chongqing, China

**Keywords:** KIRC, sphingolipid gene, prognostic model, risk score, tumor

## Abstract

This study aimed to explore underlying mechanisms by which sphingolipid-related genes play a role in kidney renal clear cell carcinoma (KIRC) and construct a new prognosis-related risk model. We used a variety of bioinformatics methods and databases to complete our exploration. Based on the TCGA database, we used multiple R-based extension packages for data transformation, processing, and statistical analyses. First, on analyzing the CNV, SNV, and mRNA expression of 29 sphingolipid-related genes in various types of cancers, we found that the vast majority were protective in KIRC. Subsequently, we performed cluster analysis of patients with KIRC using sphingolipid-related genes and successfully classified them into the following three clusters with significant prognostic differences: Cluster 1, Cluster 2, and Cluster 3. We performed differential analyses of transcription factor activity, drug sensitivity, immune cell infiltration, and classical oncogenes to elucidate the unique roles of sphingolipid-related genes in cancer, especially KIRC, and provide a reference for clinical treatment. After analyzing the risk rates of sphingolipid-related genes in KIRC, we successfully established a risk model composed of seven genes using LASSO regression analysis, including SPHK1, CERS5, PLPP1, SGMS1, SGMS2, SERINC1, and KDSR. Previous studies have suggested that these genes play important biological roles in sphingolipid metabolism. ROC curve analysis results showed that the risk model provided good prediction accuracy. Based on this risk model, we successfully classified patients with KIRC into high- and low-risk groups with significant prognostic differences. In addition, we performed correlation analyses combined with clinicopathological data and found a significant correlation between the risk model and patient’s M, T, stage, grade, and fustat. Finally, we developed a nomogram that predicted the 5-, 7-, and 10-year survival in patients with KIRC. The model we constructed had strong predictive ability. In conclusion, we believe that this study provides valuable data and clues for future studies on sphingolipid-related genes in KIRC.

## Introduction

Kidney cancer is a common human malignant tumor associated with high morbidity and mortality rates. Approximately 430,000 new cases and 180,000 deaths are recorded every year (Sung et al., 2021). Clear cell renal cell carcinoma (ccRCC), also known as kidney renal clear cell carcinoma (KIRC), is the main subtype (Frew and Moch, 2015; Capitanio et al., 2019). Current treatment methods for renal cell carcinoma (RCC) include radical nephrectomy, postoperative adjuvant therapy, radiotherapy, chemotherapy, targeted therapy, and immunotherapy (Lam et al., 2005; Flippot et al., 2018). The early clinical symptoms of kidney cancer are not obvious, prognosis is poor at the late stage, and metastatic RCC shows strong resistance to traditional radiotherapy and chemotherapy. Over time, molecular targeted drug therapy and immunotherapy have improved the therapeutic landscape of patients with advanced KIRC (Rini et al., 2019). Regrettably, many patients eventually develop drug resistance, and the tumor progresses (Au et al., 2021). Therefore, we must continue researching new RCC treatment methods and identifying new predictors.

Sphingolipids are biologically active lipids widely present in eukaryotic cells that maintain the barrier function and fluidity of cell membranes (Hannun and Obeid, 2008). Members of the sphingolipid family are widely involved in cancer cell growth, migration, invasion, and other biological processes (Hannun and Obeid, 2018). Sphingolipids and related derivatives have been extensively studied as potential therapeutic targets in cancer research. Important sphingolipid molecules are mainly ceramide (Cer), sphingosine (Sph), and sphingosine 1-phosphate (S1P). Among them, Cer and Sph mainly cause cell cycle arrest and promote cell apoptosis, while S1P mainly promotes cell survival. The balance between pro-apoptotic Cer/Sph and pro-survival S1P determines cell fate, known as “sphingolipid-rheostat”, and regulating this balance has been considered a new strategy for tumor therapy (Haass et al., 2015; Ogretmen, 2018). In addition, dihydroceramide accumulates in cells by inhibiting ceramide desaturation. This phenomenon is related to the regulation of autophagy, especially cancer cell death induced by autophagy (Jiang and Ogretmen, 2014). Glucosylceramide synthase (GCS) is involved in sphingolipid metabolism, and its role in regulating doxorubicin resistance in breast cancer cells has been demonstrated (Zhang et al., 2009; Baran et al., 2011). Sphingolipids and related derivatives have a unique influence on cancer progression (Samaha et al., 2019). Therefore, we aimed to determine the relationship between sphingolipids and RCC, and further study the role of sphingolipids in reversing KIRC resistance.

We screened 29 sphingolipid pathway genes using Gene Set Enrichment Analysis (GSEA). Based on the TCGA database, we obtained CNV, SNV, and mRNA expression data for 32 cancers. Next, the data were processed, and related research on sphingolipid gene methylation was carried out. Following that analysis, we performed correlation scoring and cluster analysis of the data. Additional research, such as drug sensitivity correlation analysis and immune cell transcription factor correlation analysis, was also conducted based on cluster analysis. After risk rate evaluation and least absolute shrinkage and selection operator (LASSO) Cox regression analysis, we ultimately selected seven genes including SPHK1, CERS5, PLPP1, SGMS1, SGMS2, SERINC1, and KDSR by application of univariate and multivariate Cox analyses, and produced a model in the form of a nomogram to evaluate prognosis of patients with KIRC. Therefore, we believe this study can provide valuable data and information for future sphingolipid cancer research.

## Materials and Methods

### Data Acquirement and Pan-Cancer Analysis

The 29 sphingolipid pathway-related genes were obtained from the GSEA package on the WikiPathways website (https://www.gsea-msigdb.org/gsea/index.jsp) (Mootha et al., 2003; Subramanian et al., 2005). First, we downloaded the TCGA dataset (https://portal.gdc.cancer.gov) to acquire CNV, SNV, and changes in expression levels. These genetic data are from 32 types of cancers. Data were analyzed using Perl language as well as R Studio. Then, the Toolbox for Biologists (TBtools) was used to visualize the data (Chen et al., 2020). We collected RNA-Seq transcriptome data from TCGA and downloaded the related clinicopathological data (Supplementary Table S1). These data included 539 tumor samples and 72 normal samples. In addition, based on the obtained data, we analyzed the relationship between CNV in sphingolipids and cancer mRNA expression on the Gene Set Cancer Analysis (GSCALite) website (Liu et al., 2018) and drew related heat maps.

### Assessment of Sphingolipid Gene Methylation on Survival of Kidney Renal Clear Cell Carcinoma

To determine the impact of sphingolipid gene methylation in KIRC, we first analyzed the relationship between sphingolipid gene methylation and 14 cancers (including BRCA, PRAD, and LUAD) on the GSCALite website and drew related heat maps. Based on these results, we analyzed the association between sphingolipid gene methylation and cancer mRNA expression. To obtain statistically significant results, we selected data with *p* < 0.05. In the resulting figures, the association is displayed as a solid sphere where the size of the sphere represents the relevance, and the color shows the increase and decrease in mRNA expression. Finally, we focused on the impact of sphingolipid gene methylation on KIRC survival. We analyzed DFI, DSS, OS, and PFS separately, and the results are displayed by related heat maps (*p* < 0.05).

### Cluster Analysis Based on Sphingolipid Score

The previous dataset showed changes that were statistically significantly different. Consequently, we constructed a sphingolipid scoring model based on mRNA expression to illustrate the differences between the samples. In view of the above, we evaluated the enrichment fraction of sphingolipid pathway genes through single-sample Gene Set Enrichment Analysis (ssGSEA). Differential analysis was performed using the “gplots” package in RStudio, and the heatmap of cluster analysis results was generated with the “pheatmap” package. The mRNA expression levels in normal tissues were initially analyzed and the mRNA expression status in tumor tissues was divided into three groups: active sphingolipid-related genes (Cluster 1 or C1), inactive sphingolipid-related genes (Cluster 2 or C2), and general sphingolipid-related genes (Cluster 3 or C3). In addition, we created a violin chart using the RStudio “ggpubr” package. This analysis specified the gene enrichment of each of the three clusters and further illustrated the expression levels. Finally, we created a heat map to express the association between the first two clusters and the clinicopathological characteristics of patients with KIRC using the “pheatmap” in RStudio. A result of *p* < 0.05 indicated that the difference was statistically significant.

### Regulon Analysis

The entire transcriptional regulatory network was rebuilt using the R package “RTN.” To discover the association between regulators and possible targets, we used mutual information analysis and the Spearman rank-order correlation method. The associations with FDR >0.00001 had to be deleted so we applied permutation analysis. It was also necessary to clarify the connection between variable associations. Using a bootstrapping strategy, and after thousands of resamplings, a consensus bootstrap result exceeding 95% was obtained. Next, we calculated the DPI-filtered regulatory network and utilized data processing inequality filtering. The two-sided GSEA allowed evaluation of the single regulon activity. Finally, we used the “MIBC_regact” package to draw related heat maps.

### Drug Sensitivity Analysis Based on the Genomics of Drug Sensitivity in Cancer Database

We selected 12 drugs from the 266 drugs listed in the 2019 GDSC database (https://www.cancerrxgene.org/). The “pRRopheticl” package was used to build a ridge regression model, which could provide an estimate of the half maximum inhibitory concentration (IC 50) of the drugs in the three clusters (Aykul and Martinez-Hackert, 2016). The prediction accuracy was evaluated based on a 10-fold cross-validation of the GDSC training set. All parameters except “combat” and “allSoldTumours” tissue patterns were set to default values, and the expression level of repeated genes was adjusted to the average value. Finally, we used the “ggplot2” and “cowplot” packages to draw box plots. Statistical significance was set at *p* < 0.05. Additionally, based on the drug sensitivity data of GDSC and CTRP on the GSCALite website, we analyzed 23 sphingolipid pathway genes and their relationships.

### Classic Cancer-Related Genes and Histone Modifications

To determine the possible regulatory mechanism of sphingolipid pathway genes in KIRC, we detected the expression of cancer-related genes in the three clusters, and the results were expressed in the form of heatmaps. This method utilized the “string,” “gplots,” “gird,” and “pheatmap” packages. Correspondingly, one-way ANOVA was applied to compare the expression levels of cancer-related genes in different clusters. Statistical significance was set at *p* < 0.05. Sirtuin (SIRT) and histone deacetylase (HDAC) not only participate in histone modification, but also play a critical role in regulating the production of biologically active lipids. We used the same method to demonstrate the differences in the expression of sirtuin (SIRT) and HDAC among the three sphingolipid-related clusters.

### Correlation Between Sphingolipid Score and Immune Cell Infiltration

We obtained 29 immune-related gene sets from TCGA and used ssGSEA to quantify them. Subsequently, we drew a heat map of the correlation between sphingolipid-related genes and immune cell infiltration by using the “ggplot2” and “dplyr” packages in RStudio. The Spearman correlation coefficient was utilized for statistical analysis. Based on the results, we used the “ggstatsplot,” “data.table,” “dplyr,” “tidyr,” and “ggplot2” packages in RStudio to analyze and visualize the association between sphingolipid scores and immune substances. Finally, we used the “ggscatterstats” package to create a scatter plot showing the relationship between the type II interferon (IFN) response and the scores of the genes related to the sphingolipid pathway. Statistical significance was set at *p* < 0.05.

### Construction of a Risk Model Using Least Absolute Shrinkage and Selection Operator Cox Regression Analysis

Initially, we conducted a hazard ratio assessment. The “glmnet” package was used to perform LASSO Cox regression analysis to further determine the most valuable prognostic genes and build risk models. Next, we applied the following formula to calculate the risk score (RS) of every sample based on gene expression and coefficient values: risk score = ∑ni = 1coefi×xi, where coefi represents the coefficient and xi represents the expression value of each selected gene. The “survminer” package was used to acquire the best cutoff value and then we divided the sample into two different groups: high-risk and low-risk. The “Kaplan-Meier survival” package in R was used to calculate the survival curves for the two groups. Subsequently, we used the “survivalROC” package in R to generate receiver operating characteristic (ROC) curves. Moreover, we used the “timeROC” R package to calculate the area under the curve (AUC) value of each model. Finally, we analyzed the correlation between the clinicopathological characteristics of patients with RS and KIRC using heat maps based on these models. Due to the massive amount of patient Nx data in the TCGA dataset, no stage N data were obtained. Statistical significance was set at *p* < 0.05.

### Construction of a Nomogram to Predict Patient Prognosis Kidney Renal Clear Cell Carcinoma

Initially, we obtained relevant immunohistochemical information from the Human Protein Atlas (HPA). The correlation between patient age, tumor stage, tumor grade, tumor size (T), tumor metastasis (M), and RS in the model was determined using univariate and multivariate Cox regression analyses. Finally, we used the “rms” package to design a nomogram based on the Cox regression analysis results and clinical characteristics. Ultimately, we were able to evaluate the survival probability of patients with KIRC using the nomogram.

### Kidney Renal Clear Cell Carcinoma Tissue Samples

From January 2022 to April 2022, renal cancer tissue and adjacent paired normal tissue were resected from six patients in our hospital who did not receive other treatments after surgery, and the histopathological subtype was identified as KIRC by a pathologist. This study was approved by the Medical Ethics Committee of our hospital, and the patients gave informed consent. After the pathological specimens were excised, the samples were cut into small pieces, an RNA protective agent was added, and the samples were finally stored in a −80°C refrigerator. In this study, we extracted RNA from these six pairs of pathological tissues and detected the mRNA expression of SGMS2 in them.

The primer sequences used in this manuscript are as follows: SGMS2 (forward, 5′-CTT​AGC​CCT​CCA​CTC​CC-3′ and reverse, 5′-CAG​AAT​CTG​CGT​CCC​AC-3′) and GAPDH (forward, 5′- GGA​GCG​AGA​TCC​CTC​CAA​AAT-3′ and reverse, 5′-GGC​TGT​TGT​CAT​ACT​TCT​CAT​GG-3′).

### *In Vitro* Cell Experiments Targeting Sphingomyelin Synthase 2

In this study, the human KIRC cell lines 786-O and ACHN cells were purchased from the Cell Bank of the Chinese Academy of Sciences. All cells were cultured according to the manufacturer’s protocol. 786-O cells were cultured in RPMI 1640 medium containing 10% fetal bovine serum, and ACHN cells were cultured in high-glucose Dulbecco’s Modified Eagle Medium (DMEM) containing 10% fetal bovine serum. First, we cultured 786-O and ACHN renal cancer cell lines in a laboratory incubator using cell culture techniques. Subsequently, we established SGMS2-overexpressing KIRC cell lines by transfecting 786-O and ACHN cells with 10 ug/mL plasmid (GenePharma, Shanghai) using Lipofectamine 3000 reagent (Invitrogen, California). Finally, we performed CCK8 cell proliferation experiments and Transwell cell migration experiments in 786-O and ACHN renal cancer cell lines.

## Results

### Widespread Mutations of Sphingolipid Pathway Genes in 32 Cancers

**FIGURE 1:**
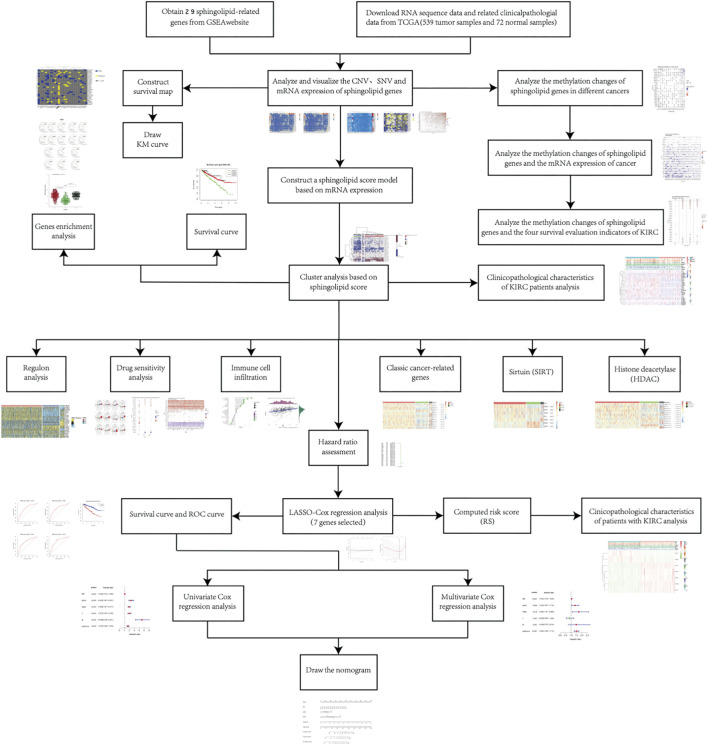
The flowchart illustrates the research process.

**FIGURE 2:**
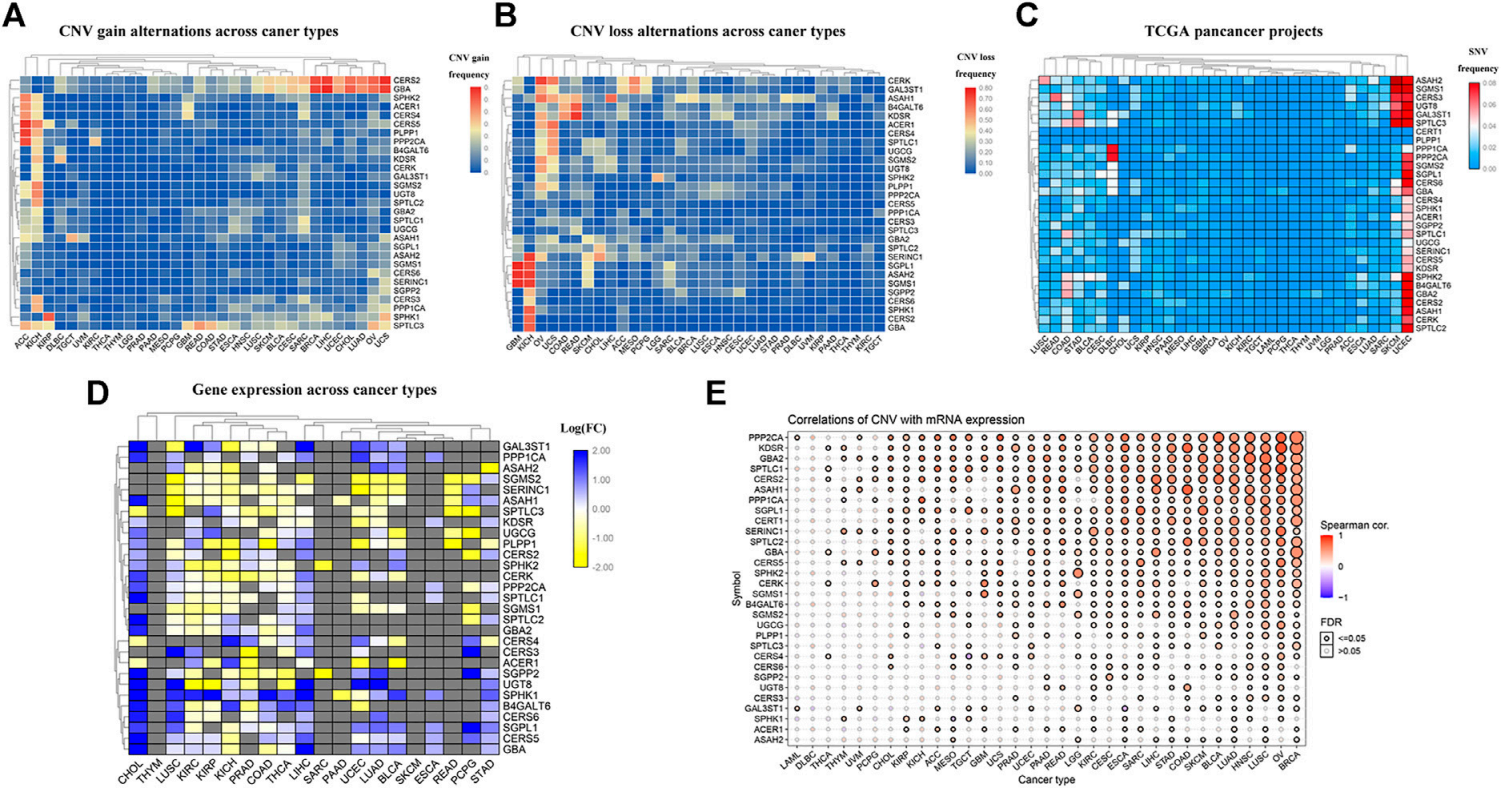
Widespread genetic mutations of sphingolipid pathway genes. **(A)** CNV gains of sphingolipid pathway genes in 32 types of cancers. The adjacent color block represents the frequency of mutations, the red color bar represents a high mutation frequency, and the blue color bar represents low mutation frequency. **(B)** CNV losses in sphingolipid pathway genes. The adjacent color block represents the frequency of mutations, the red color bar represents a high mutation frequency, and the blue color bar represents a low mutation frequency. **(C)** SNV in sphingolipid pathway genes. The adjacent color block represents the frequency of mutations, the red color bar represents a high mutation frequency, and the blue color bar represents a low mutation frequency. **(D)** mRNA expression of sphingolipid pathway genes. The blue color bar represents increased mRNA expression and the yellow color bar represents decreased mRNA expression. **(E)** Correlations of CNV and mRNA expression. A solid sphere indicates that the result is statistically significant (*p* < 0.05), the size of the sphere indicates the degree of correlation, and the larger the sphere, the higher the degree of correlation.

At the outset, we created a flowchart of the study to illustrate each step (Figure 1). The CNV and the 32 different types of cancer were obtained from the GSEA website and referred to the TCGA dataset. We observed that CNV and SNV were present in genes related to the sphingolipid pathway in most cancer types; however, almost no CNV was gained or lost in Thymoma. Ceramide Synthase 2 and GBA are widely acquired by CNV in various types of cancers (Figures 2A,B). High-frequency SNVs were observed in DLBC, SKCM, and UCEC. In contrast, the SNV frequency was lower in the Thymoma, THCA, and PRAD groups (Figure 2C). We used a log2 (fold change) to depict the ratio of gene expression status in cancer tissues to that in normal tissues. The results showed changes in sphingolipid gene expression in different cancer types. Gene expression levels in cancer tissues changed significantly when compared to those in normal tissues (Figure 2D). Furthermore, the results based on the GSCALite website showed that CNV of sphingolipid-related genes could lead to increased mRNA expression in cancer (Figure 2E).

### Sphingolipid Genes are Mostly Protective in Kidney Renal Clear Cell Carcinoma

**FIGURE 3:**
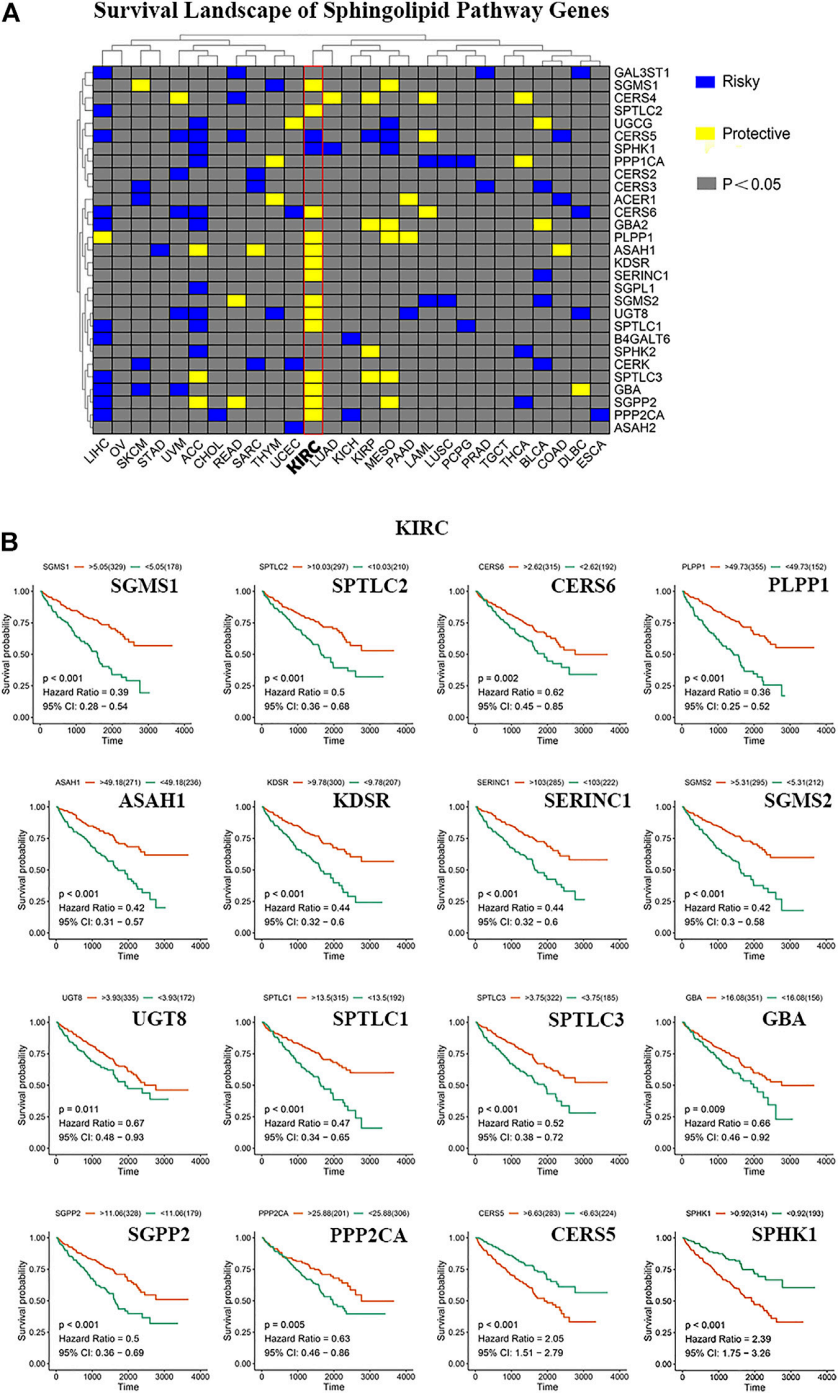
The role of sphingolipid pathway genes in cancer. **(A)** Survival map of sphingolipid genes. Blue represents risk genes and yellow represents protective genes. The gray bar represents no statistical significance. **(B)** The survival curve of sphingolipid pathway genes in kidney renal clear cell carcinoma (KIRC). We selected 16 meaningful genes based on the survival map. According to the best cutoff value, the samples are divided into high-expression groups and low-expression groups, and the associated survival curve is drawn. The orange line represents the high-expression groups, and the green line represents the low-expression groups. The abscissa represents the number of days, and the ordinate represents the survival probability.

We constructed a survival map based on the association between the patient survival rate and gene expression in TCGA (Figure 3A). If the hazard ratio (HR) was <1, the gene was considered a protective gene, and if the value was >1, the gene was considered a risk gene. Genes related to the sphingolipid pathway can both promote and inhibit the growth of cancer cells in tumors. Generally, in tumors, the expression of protective genes decreases and the expression of risk genes increases; however, we found that the three genes PLPP1, KDSR, and GBA were all protective factors of KIRC. However, these genes are also upregulated, which appears to be contradictory. The results of pan-cancer analysis indicated that most sphingolipid pathway-related genes were protective genes in patients with KIRC, whereas they were not obviously so in many other cancers. Since strong lipid metabolism, ease of transfer, and resistance to radiotherapy and chemotherapy are all peculiarities of KIRC, we will focus on the relationship between sphingolipid pathway-related genes and disease in follow-up research. Using the “survminer” package, the genes were divided into high-expression groups and low-expression groups according to the best cutoff value. We then used the Kaplan-Meier curve to represent statistically significant sphingolipid-related genes in patients with KIRC (Figure 3B). This result is consistent with the original survival map that we constructed (Figure 3A).

### Effect of Sphingolipid Gene Methylation on Survival of Kidney Renal Clear Cell Carcinoma

**FIGURE 4:**
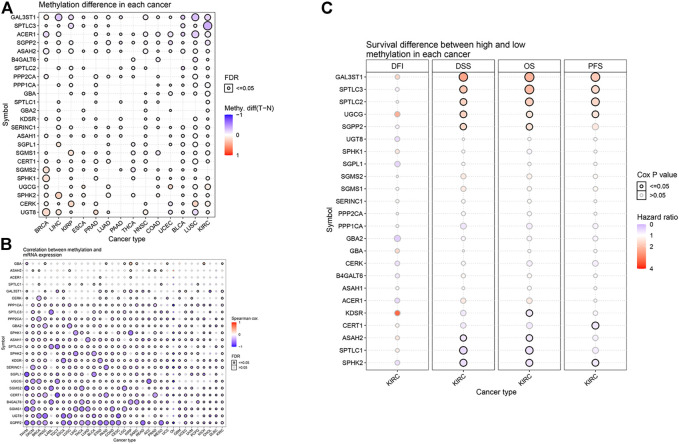
Effect of sphingolipid methylation. The solid spheres demonstrate that the result is statistically significant (*p* < 0.05), the size of the sphere shows the degree of correlation, and the larger the sphere, the higher the degree of correlation. **(A)** Widespread methylation of sphingolipid-related genes in 14 cancers. In the color bar on the right side, red represents an increase in methylation level, and blue represents a decrease level. **(B)** The relationship between sphingolipid gene methylation and cancer mRNA expression. In the color bar on the right side, red represents an increased mRNA expression, blue represents a decreased mRNA expression. **(C)** Sphingolipid gene methylation and KIRC survival evaluation index. The color bar on the right side represents the HR. Red represents a higher HR (the maximum value is 4), which means a longer life span, and gray represents a lower risk ratio (the minimum value is 0), which means insignificant change. The methylation of GAL3ST1, SPTLC3, SPTLC2, and UGCG can lead to prolongation of OS, DSS, and PFS in patients with KIRC, and SGPP2 methylation can lead to prolonged OS and DSS. The change in DFI is not statistically significant.

DNA methylation has been widely studied as an epigenetic modification in cancer. DNA methylation changes are closely related to clinicopathological characteristics and patient survival rates. Some researchers have predicted the prognosis of KIRC by analyzing the DNA methylation of Hugl-2. Based on the GSCALite website, we analyzed the relationship between sphingolipid-related gene methylation and 14 cancers (Figure 4A). The results showed that sphingolipid genes undergo extensive methylation changes in these cancers. Next, we analyzed the relationship between sphingolipid gene methylation and cancer mRNA expression (Figure 4B). The resulting heat map showed a strong correlation, and methylation of most sphingolipid genes resulted in decreased mRNA expression. However, the methylation of GBA and ASAH2 led to increased mRNA expression. Finally, we analyzed the relationship between sphingolipid gene methylation and four survival indicators (Figure 4C). The results showed that methylation of GAL3ST1, SPTLC3, SPTLC2, and UGCG could lead to prolongation of OS, DSS, and PFS in patients with KIRC, and SGPP2 methylation could lead to prolonged OS and DSS. In summary, these results indicate that methylation of sphingolipid-related genes plays a vital role in cancer progression and may have a positive influence on the survival of patients with kidney cancer.

### Cluster Analysis Based on the Scores of Sphingolipid Pathway-Related Genes

**FIGURE 5:**
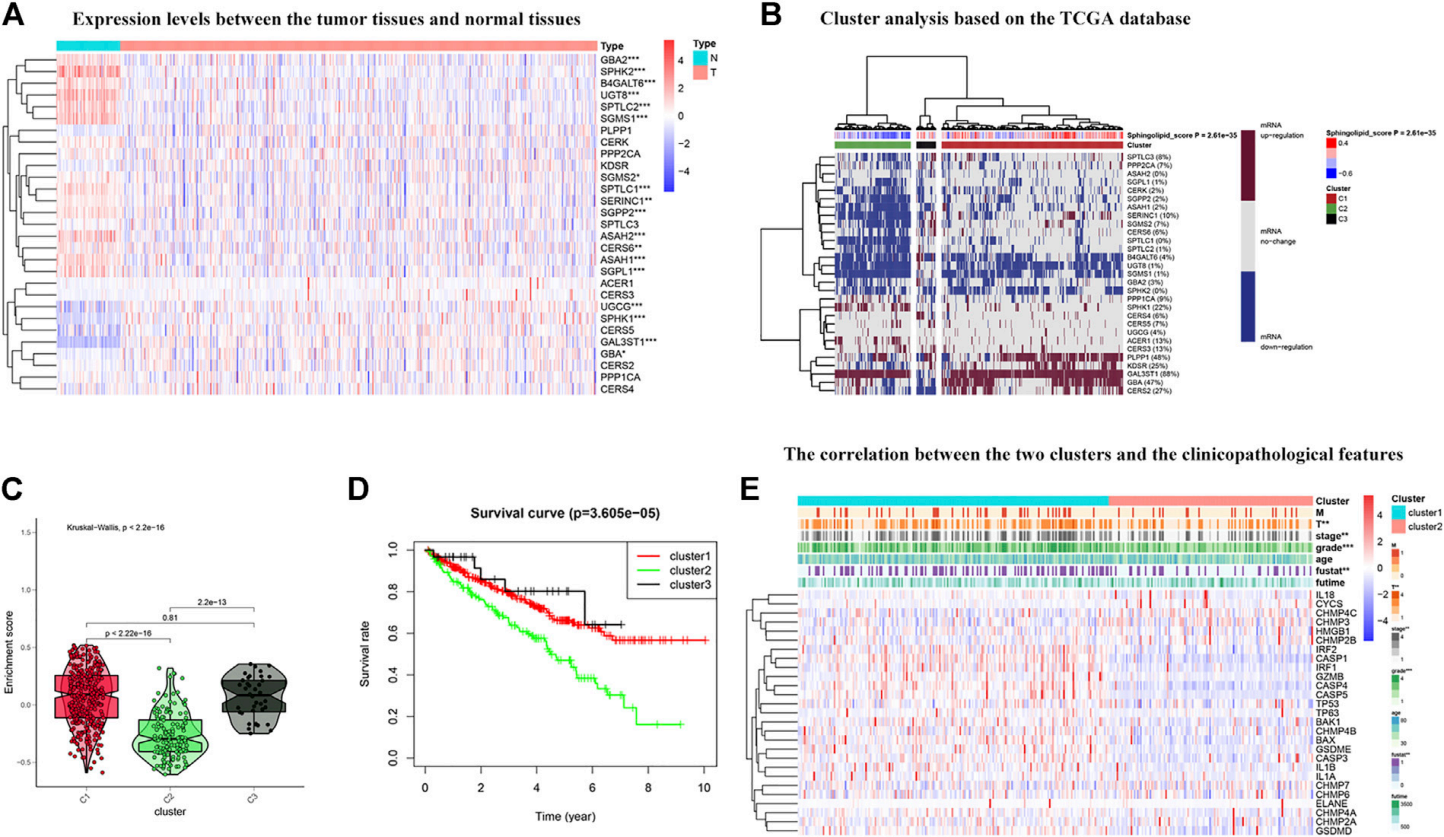
Cluster analysis based on the sphingolipid scores. **(A)** The heat map shows the difference in expression between normal tissues and tumor tissues. The color in the block on the right represents gene regulation; red represents upregulation, and blue represents downregulation. N (blue) represents the normal sample, T (red) represents the tumor sample (*:*p* < 0.05, **:*p* < 0.01, ***:*p* < 0.001). **(B)** Heat map of three clusters: active sphingolipid-related genes (Cluster 1 or C1), inactive sphingolipid-related genes (Cluster 2 or C2) and generasl sphingolipid-related genes (Cluster 3 or C3). The percentage of patients whose genes are upregulated is given on the right side of the figure. In the color bar on the right side, red represents mRNA upregulation, blue represents mRNA downregulation, and gray represents mRNA no-regulation. The four color bars represent the sphingolipid score; red represents positive values, and blue represents negative values. **(C)** The violin plot shows the enrichment scores of the three clusters. From high to low: Cluster 1, Cluster 2, and Cluster 3. The *p*-values are displayed above the clusters. **(D)** The survival curves associated with the three clusters. The survival rate of Cluster 2 is lower than that of Clusters 1 and 3. The red line represents Cluster 1, the green line represents Cluster 2, and the black line represents Cluster 3. The abscissa represents the number of years and the ordinate represents the survival probability. **(E)** The heat map shows the correlation between the two clusters and the clinicopathological features. In the color bar on the right side, red represents gene upregulation, and blue represents gene downregulation (*:*p* < 0.05, **:*p* < 0.01, and ***:*p* < 0.001).

To further explore the expression of genes related to the sphingolipid pathway in KIRC, we generated heat maps (Figure 5A). We noticed that the expression levels of these genes were significantly different between tumor and normal tissues. Next, these genes were analyzed in the KIRC univariate Cox regression analysis. High expression levels of SGMS1, SPTLC2, CERS6, PLPP1, ASAH1, KDSR, SERINC1, SGMS2, UGT8, SPTLC1, SPTLC3, GBA, SGPP2, and PPP2CA were associated with better survival rates in patients with KIRC and conversely, high expression levels of CERS5 and SPHK1 were associated with poor survival. We divided sphingolipid-related genes into three clusters based on the final sphingolipid pathway-related scores and gene expression levels. C1 represented tumor tissues with active sphingolipids, C2 represented tumor tissues with inactive sphingolipids, and C3 represented tumor tissues with normal expression of sphingolipids (Figure 5B). A violin chart intuitively shows that the order of the enrichment scores of the three clusters was C1 > C2 > C3 (Figure 5C). Next, we drew the survival curves of the three clusters to determine whether the clusters were acceptable. The OS rate of C2 patients was significantly lower than that of C1 and C3 patients, and the survival rate of C3 patients was slightly higher than that of C1 patients, a result that may have been due to fewer samples (Figure 5D). This result indicates that sphingolipid genes may be a potential protective factor. We also analyzed the association between the two clusters and clinicopathological characteristics, and the results showed that a higher sphingolipid pathway score was negatively correlated with T, grade and stage, and fustat (Figure 5E).

### Differential Expression of 23 Transcription Factor Activities in Kidney Renal Clear Cell Carcinoma Based on Cluster Analysis

**FIGURE 6:**
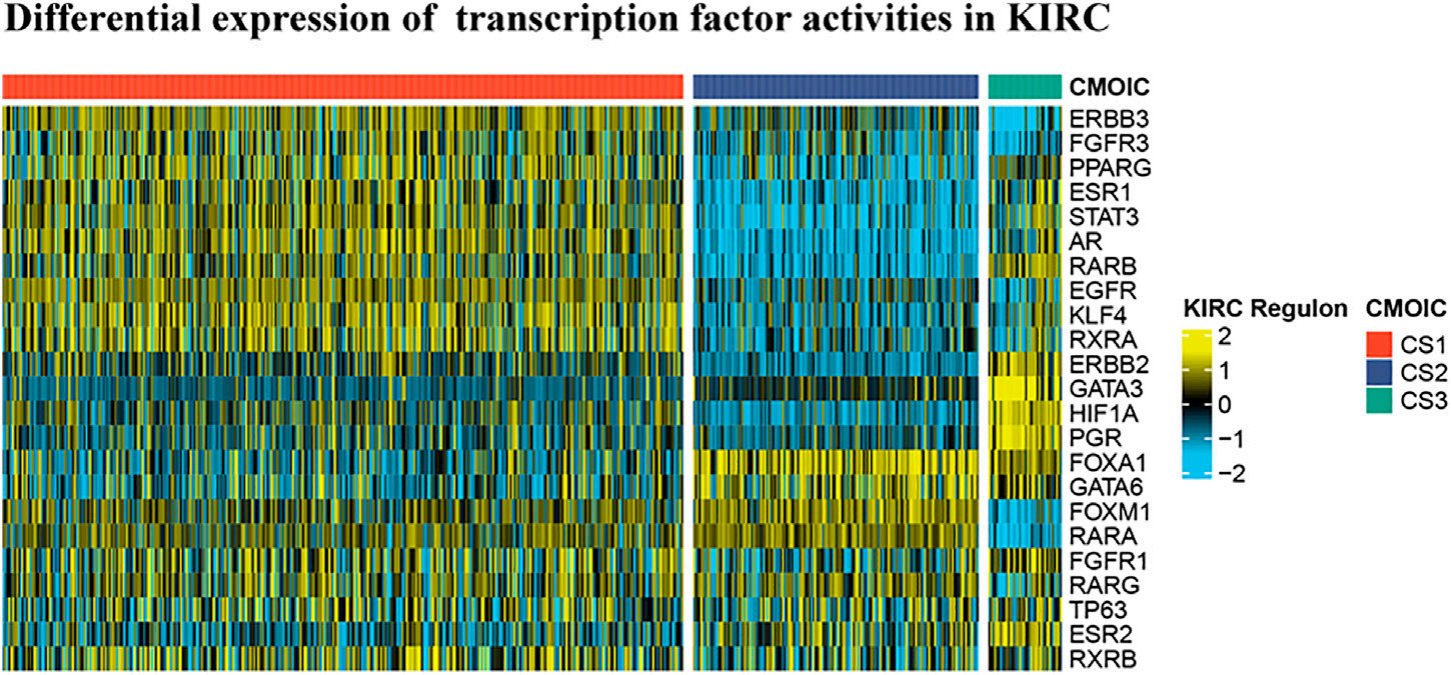
Differential expression of 23 transcription factor activities in KIRC based on cluster analysis. Based on the previous cluster analysis, the KIRC samples were divided into three clusters according to active sphingolipid genes, inactive sphingolipid genes, and normal sphingolipid genes. By analyzing the expression of 23 transcription factors in the three clusters, it is evident that significant differences exist between the three clusters.

In reference to the existing molecular classifications of RCC, and based on the three cluster samples obtained from the previous cluster analysis, we selected 23 transcription factors related to tumor progression. The results show that regulator activity is closely related to our cluster analysis, which proves that this method of dividing samples into three clusters based on sphingolipid gene activity is reliable (Figure 6). The activities of transcription factors ERBB3, FGFR3, PPARG, ESR1, STAT3, AR, RARB, EGFR, KLF4, RXRA, FOXM1, RARA, FGFR1, RARG, TP63, and RXRB in C1 were generally enhanced, and FOXA1, GATA6, FOXM1, and RARA, and the activities of RARG, TP63, ESR2, and RXRB were significantly enhanced in C2. The activity of GATA3 was not significantly changed, and the activities of the remaining 14 transcription factors were significantly weakened. The activities of ESR1, STAT3, AR, RARB, ERBB2, GATA3, HIF1A, PGR, FOXA1, GATA6, FGFR1, TP63, ESR2, and RXRB were significantly enhanced in C3, whereas the activity of PPARG was not significantly changed, and the remaining eight genes were significantly weakened. Our results showed that the activities of 23 transcription factors in the three clusters were significantly different, and the differential expression of the activities of these transcription factors may be the initiating factor for subsequent phenomena.

### Relationship Between Sphingolipid Clusters and Drug Sensitivity

**FIGURE 7:**
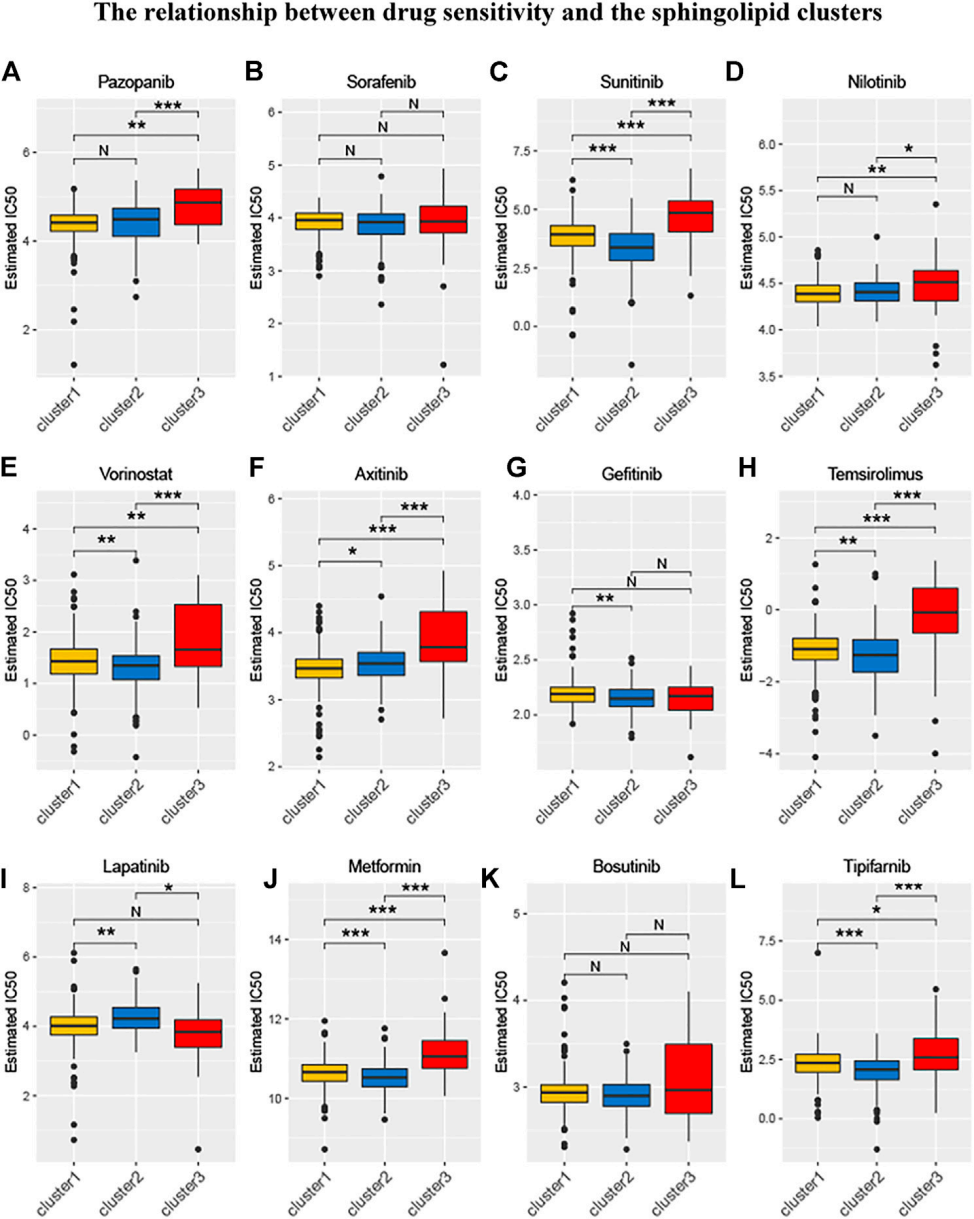
The relationship between drug sensitivity and the sphingolipid clusters. The box plots of the estimated IC50 for 12 drugs are shown in **(A–L)** for Cluster 1 (yellow), Cluster 2 (blue), and Cluster 3 (red). The 12 types of chemotherapeutic agents are pazopanib, sorafenib, sunitinib, nilotinib, vorinostat, axitinib, gefitinib, temsirolimus, lapatinib, metformin, bosutinib, and tipifarnib. The asterisk above represents the *p-*value; *p* < 0.05 was considered statistically significant (N:0.05 < *p* *:0.01 < *p* < 0.05, **:0.001 < *p* < 0.01, and ***:*p* < 0.001). The box plots indicate that drug sensitivities among the sphingolipid clusters were different.

**FIGURE 8:**
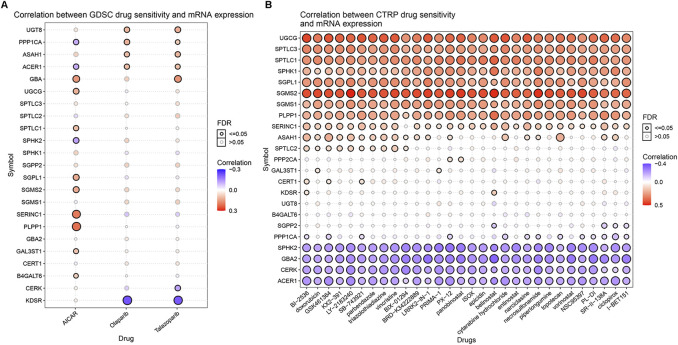
The correlations between the mRNA expression of sphingolipid genes and the drug sensitivity of GDSC and CTRP. **(A)** The correlation between mRNA expression and GDSC drug sensitivity. The solid spheres represent *p* < 0.05, and the dotted spheres represent no statistical significance. The larger the sphere, the higher the correlation. In the color bar on the right side, red represents increased mRNA expression, and blue represents decreased mRNA expression. **(B)** The correlation between mRNA expression and CTRP drug sensitivity. The solid sphere represents *p* < 0.05, and the dotted sphere represents no statistical significance. The larger the sphere, the higher the correlation. In the color bar on the right side, red represents increased mRNA expression, and blue represents decreased mRNA expression.

Relevant data for the 12 drugs were obtained from the GDSC database. These drugs include common targeted tumor drugs, especially those for kidney cancer, as well as classic drugs in tumor research, such as metformin. Sunitinib, sorafenib, and axitinib are mainly used for the targeted therapy of KIRC. Metformin is known to regulate lipid metabolism and is mainly used to treat type 2 diabetes mellitus. Metformin has been proven to exert antitumor effects in a variety of ways (Zhou et al., 2001; Hart et al., 2019). To further explore the association between these drugs and the sphingolipid pathway, we performed a drug sensitivity analysis. After the analysis, the estimated IC 50 value of the drug was obtained for every sample. A lower IC 50 value indicated increased drug sensitivity. The ridge regression model showed that the different drug sensitivities between the sphingolipid clusters were as follows: pazopanib: C2 > C3; sorafenib, no significant difference; sunitinib, C2 > C1 > C3; nilotinib, C1 > C2 > C3; vorinostat, C2 > C1 > C3; axitinib, C1 > C2 > C3; gefitinib, C2 > C1; sirolimus, C2 > C1 > C3; lapatinib, C1 > C2; metformin, C2 > C1 > C3; bosutinib, the difference was not obvious; and tipifarnib, C2 > C1 > C3 (Figure 7). In addition, we analyzed the mRNA expression of sphingolipid genes and the drug response data of GDSC and CTRP by using the GSEA website and found a strong correlation between the mRNA expression of most sphingolipid-related genes and the drug sensitivity of GDSC and CTRP (Figures 8A,B).

### Correlation Between Sphingolipid Pathway Score and Classic Cancer-Related Genes or Immune Cell Infiltration

**FIGURE 9:**
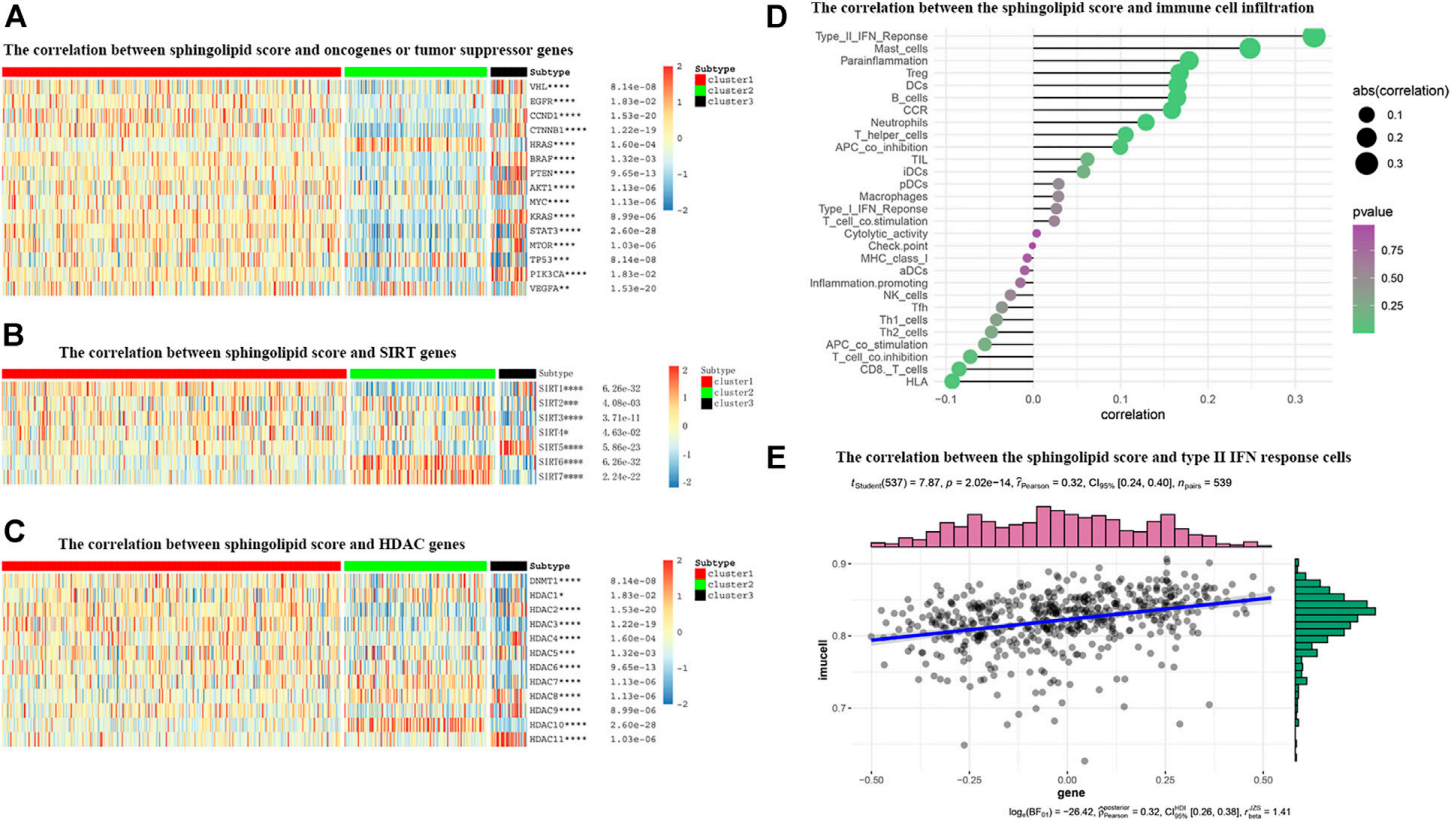
The correlations between the sphingolipid score and the classical cancer-related genes or immune cell infiltration. **(A–C)** The heatmap shows the sphingolipid score is associated with other signaling pathways in KIRC. **(A)** The interrelation with oncogenes and tumor suppressor genes. **(B)** The interrelation with sirtuin family genes. **(C)** The interrelation with histone deacetylase (HDAC) family genes. The statistical method used in **(A–C)** is “ANOVA” (*p < 0.05, **p < 0.01, ***p < 0.005, and ****p < 0.001). **(D)** The plot shows the correlation between the sphingolipid score and immune cell infiltration. On the right side of the plot, the area of the sphere represents the degree of abs (correlation) and the color indicates the *p*-value. **(E)** The scatter diagram shows the correlation between the sphingolipid score and type II IFN response cells. The sphingolipid score is positively correlated with the infiltration of type II IFN response cells.

We drew a heat map to show the differential expression of cancer-related genes in the three sphingolipid pathway clusters, and noticed that the oncogenes CCND1, BRAF, AKT1, MYC, KRAS, MTOR, PIK3CA, and VEGFA were present. The data showed that the expression level in C1 was significantly higher than that in C2. The expression levels of the tumor suppressor genes PTEN and VHL in C1 were significantly higher than those in C2. The expression level of the oncogene HRAS in C2 was significantly higher than that in C1 (Figure 9A). Among these genes, mutations in the VHL gene can cause the accumulation of HIF-1α and HIF-2α, thereby promoting tumorigenesis, and the occurrence of KIRC is strongly associated with VHL gene mutations. This indicates that the better prognosis associated with the C1 cluster may be related to the overexpression of tumor suppressor genes, and the activation of tumor suppressor genes in C1 may be more important than the activation of oncogenes. In addition, the expression of VHL, CTNNB1, BRAF, PTEN, AKT1, KRAS, MTOR, and PIK3CA increased significantly, and the expression of HRAS, MYC, STAT3, and TP53 decreased significantly in C3, the sphingolipid genes normal expression group. The expression of oncogenes and tumor suppressor genes in C3 was both increased and decreased. The prognosis of C3 was better than that of C1 and C2. This result indicates that the influence of sphingolipid genes, oncogenes, and tumor suppressor genes on prognosis is a comprehensive result, and further exploration is needed in the future.

Many studies have shown that SIRTs play an important role in tumorigenesis. Recent studies have shown that the absence of SIRT1 can cause sphingomyelin to accumulate in cells, and in different cancer types or under different experimental conditions, SIRT can act as an oncogene or a tumor suppressor gene. Our study showed that the expression levels of SIRT2, SIRT6, and SIRT7 in the sphingolipid pathway gene-inactive group were remarkably higher than those in the sphingolipid pathway gene-active group. In contrast, the expression levels of SIRT1, SIRT3, and SIRT5 in the sphingolipid pathway gene-inactive group were remarkably reduced (Figure 9B). Wei et al. analyzed mouse stem cells lacking SIRT1, and demonstrated that SIRT1 can influence sphingolipid metabolism via deacetylation of the c-Myc transcription factor. In brief, these results indicate that SIRTs are closely associated with the sphingolipid pathway and may play a synergistic role in promoting or inhibiting several processes in the progression of KIRC.

HDAC catalyzes the removal of acetyl groups from histone and non-histone lysine residues, and regulates gene transcription. This type of event is closely associated with tumorigenesis and M. HDAC inhibitors have been shown to inhibit tumor development. We noticed that the expression levels of HDAC1, HDAC5, HDAC7, HDAC8, and HDAC10 in the sphingolipid pathway gene-inactive group were significantly higher than those in the sphingolipid pathway gene-active group. On the other hand, the expression levels of HDAC2, HDAC3, and HDAC9 in the sphingolipid pathway gene-inactive group were reduced (Figure 9C). As HDAC10 is almost exclusively expressed in the sphingolipid pathway gene-inactive group, the use of HDAC10 inhibitors may be more useful for patients with inactive sphingolipid pathway genes. These results can provide new directions and ideas for precision treatment of tumors in the future.

The role of the tumor microenvironment (TME) in cancer progression cannot be ignored. Immune cells can infiltrate tumors or affect the whole-body environment to limit tumor cell metastasis or play a role in promoting tumor growth. Sphingolipids and related bioactive lipids are inextricably linked to changes in the TME. To study the correlation between the sphingolipid production pathway and immunity in patients with KIRC, we analyzed the correlation between sphingolipid score and immune cell infiltration (Figure 9D). We discovered that the infiltration of type II IFN-responsive cells, mast cells, and HLA were positively correlated, whereas infiltration of CD8 cells was negatively correlated. Based on the correlation between the sphingolipid score and type II IFN-responsive cells, we also drew a scatter plot, and the results were consistent with previous studies, i.e., there was a strong positive correlation among them (Figure 9E).

### Construction of a Risk Model Using the Least Absolute Shrinkage and Selection Operator Cox Regression Analysis

**FIGURE 10:**
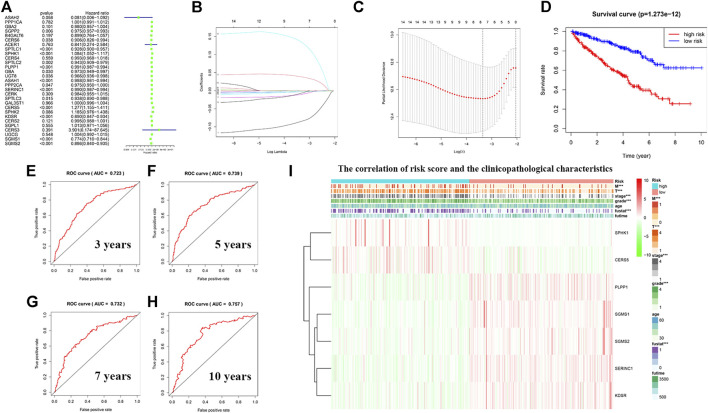
Construction of a risk model using least absolute shrinkage and selection operator (LASSO) Cox regression analysis. **(A)** The forest plot shows the resulting hazard ratios of 29 sphingolipid pathway genes in KIRC. **(B)** The LASSO coefficient profiles of sphingolipid pathway genes in KIRC. **(C)** The distribution and median value of the risk scores, using LASSO Cox regression analysis to screen out seven genes. **(D)** Survival curve drawn according to the model. The overall survival rate of patients in the high-risk group was significantly lower than that of patients in the low-risk group. Red represents the high-risk group, blue represents the low-risk group. **(E–H)** ROC curves associated with 3, 5, 7, and 10 years **(I)** Correlation heatmap of risk scores and clinicopathological characteristics. The red bar represents gene upregulation, and the green bar represents gene downregulation (***p < 0.001).

To avoid selection bias, we initially analyzed the hazard ratios of 29 sphingolipid-related genes, and presented the results in a forest map (Figure 10A). To determine whether sphingolipid-related genes can be used to construct a model for estimating the survival rate of patients with KIRC, we applied LASSO Cox regression analysis to test the 29 genes and eventually selected seven genes to construct a risk-scoring model (Figures 10B,C). Patients were divided into high-risk and low-risk groups according to their RS values. The OS rate of patients in the high-risk group was significantly lower than that of the low-risk group (Figure 10D). ROC curve analysis was used to test the predictive effect of the new survival model on the prognosis of these patients. The following are the areas under the ROC curves for the survival models: the 3-year survival rate prediction was 0.723; the 5-year survival rate prediction was 0.739; the 7-year survival rate prediction was 0.732; and the 10-year survival rate prediction was 0.757. The AUC values were all greater than 0.7, thereby indicating that our model had a high predictive value (Figures 10E–H). Next, we performed further statistical tests on the differences between the risk subgroups and created a heat map to visualize the correlation between RS and clinical data (Figure 10I). The results showed that our risk model was related to tumor M, T, stage, grade, and fustat. Patients in the high-risk group often have advanced histological grades and are in advanced clinical stages.

### Predicting the Outcome of Patients With Kidney Renal Clear Cell Carcinoma Using a Nomogram

**FIGURE 11:**
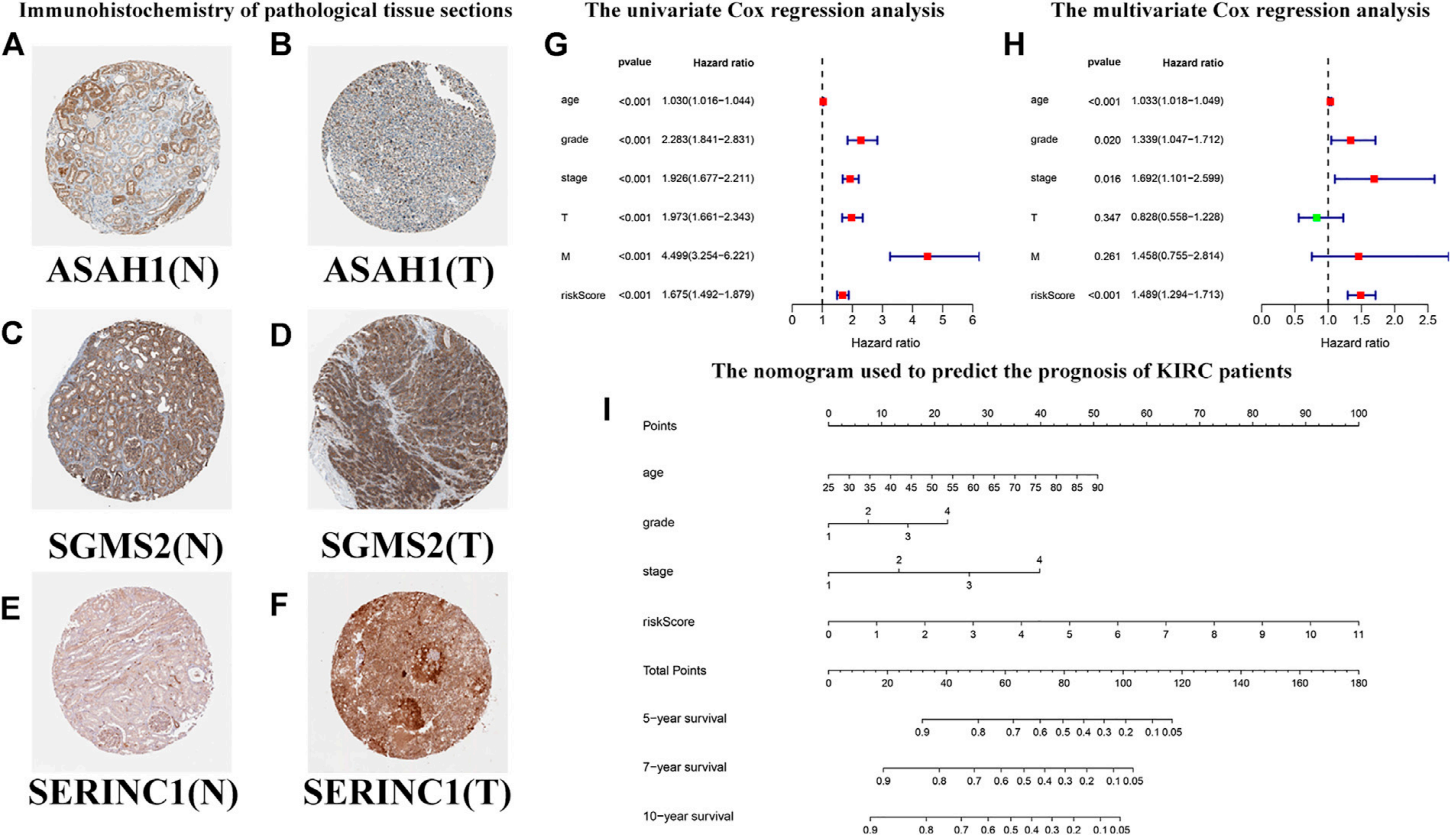
Predicting the outcomes of patients with KIRC using a nomogram. **(A–F)** Immunohistochemistry of pathological tissue sections. “N” stands for normal tissue morphology, “T” stands for KIRC tumor morphology. **(G)** Univariate Cox regression analysis of the correlation with risk scores (RSs), clinicopathological parameters (age, grade, stage, T, and M), and overall survival (OS) of patients with KIRC. The forest plot shows that the age, tumor grade, stage, T, M, and RS are correlated with the OS of the patients (*p* < 0.05). **(H)** Multivariate Cox regression analysis of the correlation with RS, clinicopathological parameters, and the OS of patients with KIRC. The forest plot reveals that patient age, grade, stage, and RS are independent risk factors correlated with OS (*p* < 0.05). **(I)** The nomogram incorporates RS, age, grade, and stage, which can be used to predict the outcome of patients with KIRC. The first to fifth lines represent the points, patient age, tumor grade, tumor stage, and RS. The total score in the sixth row is the sum of the scores for each item from the first to fifth lines. The 5-, 7-, and 10-year survival rates are predicated based on the total score.

Based on previous HR analysis results, we selected 16 genes with statistical significance. We obtained immunohistochemical information from the HPA website and certified their gene expression results at the protein level (Figures 11A–F). We performed univariate Cox regression analysis of RS and other clinicopathological characteristics (Figure 11G). The resulting forest plot indicates the relationship between the clinicopathological characteristics and the OS rate of the patient. Patient age, tumor grade, stage and T, M, and RS were all included. Multivariate Cox regression analyses showed that these clinicopathological characteristics were independent risk factors related to OS (Figure 11H). Based on the nomogram of the risk model (Figure 11I), the second to eighth rows represent patient age, tumor grade, tumor stage, RS, total score, and 5-year, 7-year, and 10-year survival rates, respectively. The total score in the sixth row is the sum of the scores of the items in the first through fifth rows. The 5-year, 7-year, and 10-year survival rates were predicted on the basis of the total score. For example, if the total score was 80, the 5-year survival rate was approximately 0.5.

### *In Vitro* Cell Experiments to Explore the Biological Function of Sphingomyelin Synthase 2 in Kidney Renal Clear Cell Carcinoma

**FIGURE 12:**
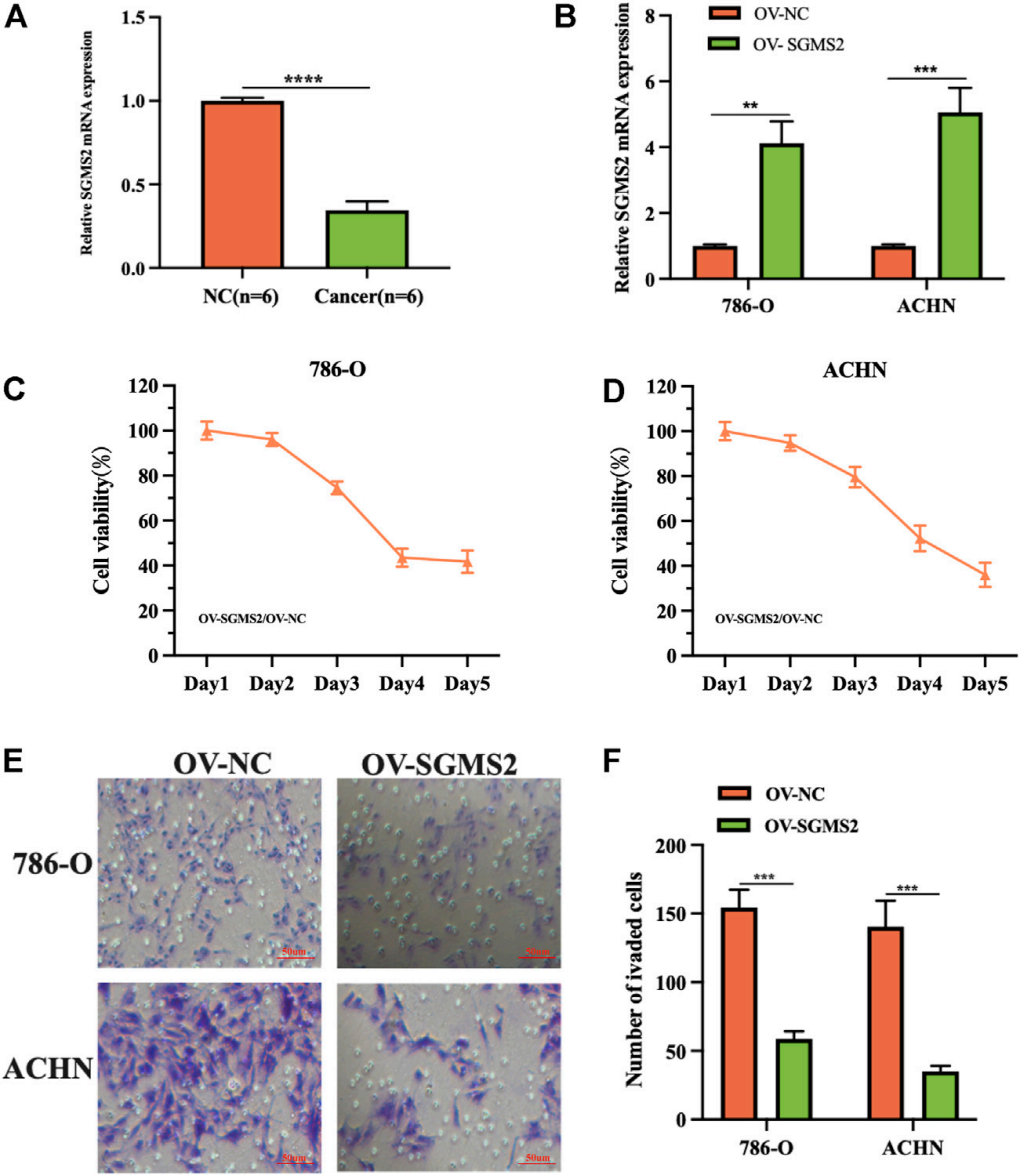
Laboratory experiments explored the expression and biological role of SGMS2 in KIRC. **(A)** The histogram of the data of RT-PCR assay detecting SGMS2 mRNA expression in six pairs of KIRC pathological tissues. **(B)** The histogram was obtained by RT-PCR experiment to detect the transfection efficiency data of SGMS2 plasmid. **(C,D)** Line graphs based on CCK8 cell proliferation assay data after overexpression of SGMS2 in 786-O and ACHN cell lines. **(E,F)** Light microscopy images of Transwell cell migration assays after overexpression of SGMS2 in 786-O and ACHN cell lines, and a corresponding histogram. **p < 0.01, ***p < 0.001, ****p < 0.0001.

**FIGURE 13:**
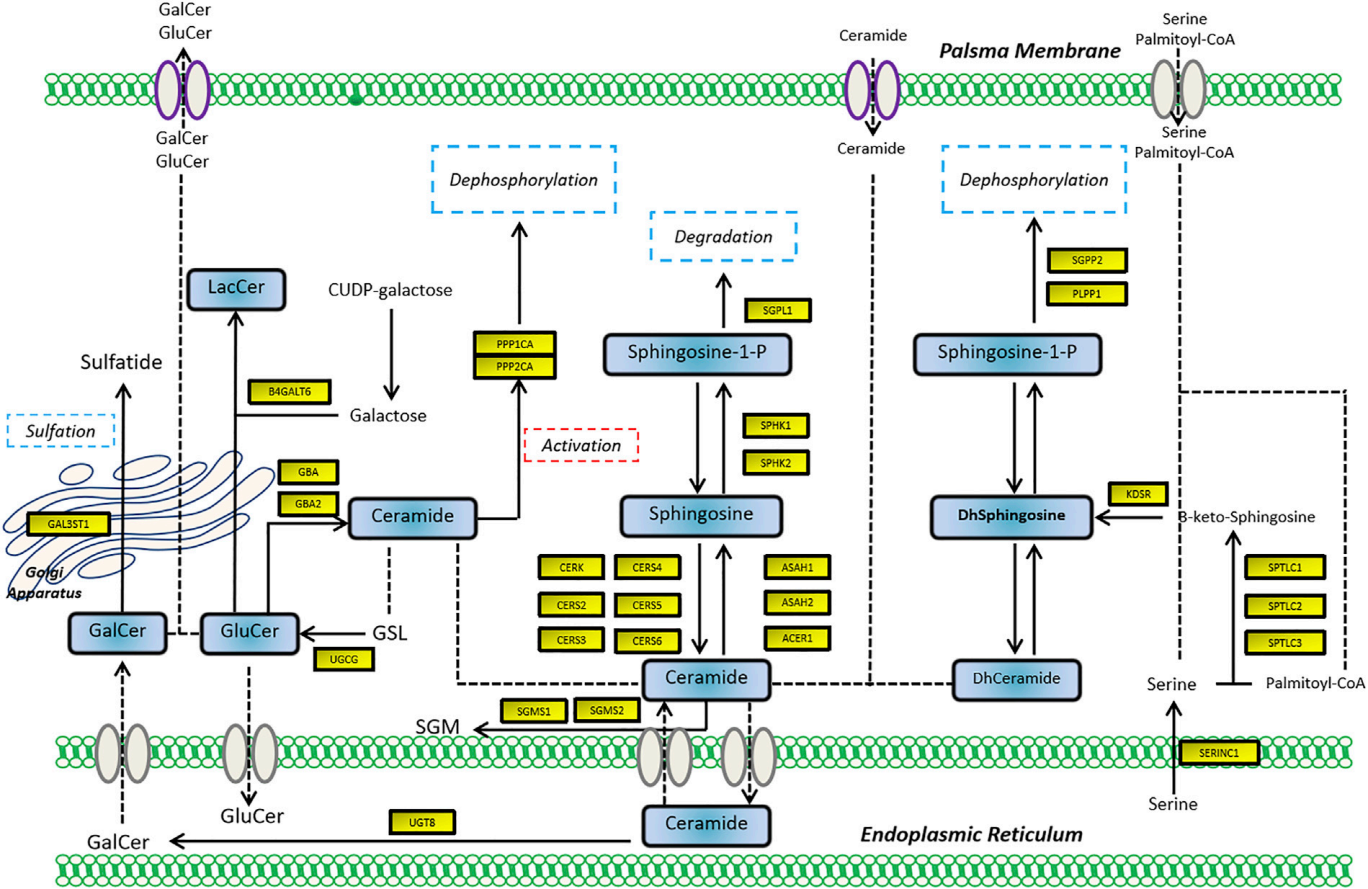
The schematic diagram shows the biological mechanism of the sphingolipid-related genes involved in this study in human cells.

To assess the expression of SGMS2 in KIRC, we used clinicopathological tissue for probing. The results of RT-PCR showed us that the expression of SGMS2 in KIRC tissues was significantly lower than that in normal kidney tissues (Figure 12A). To further explore the biological role of SGMS2 in KIRC, we established 786-O and ACHN cell lines overexpressing SGMS2 using plasmid transfection technology, and verified the plasmid transfection efficiency by RT-PCR (Figure 12B). The results confirm that we have completed this step. Subsequently, the results of CCK8 experiments showed that overexpression of SGMS2 could significantly inhibit the proliferation of 786-O and ACHN cell lines (Figures 12C,D). The Transwell cell migration assay results showed that overexpression of SGMS2 could significantly inhibit the migration of 786-O and ACHN cell lines (Figures 12E,F). This suggests that SGMS2 acts as a tumor suppressor gene in KIRC progression. Finally, to show the readers the sphingolipid-related genes involved in this study more clearly, we especially draw a schematic diagram reflecting the biological roles of sphingolipid-related genes (Figure 13).

## Discussion

Sphingolipids play an important role in maintaining membrane barrier function and fluidity (Gault et al., 2010), and affect cell signal transduction by acting as secondary messengers and regulating various biological processes (Dressler et al., 1992). In the past few years, many researchers have identified and cloned almost all the major metabolic enzymes that regulate the relative abundance of sphingolipids. As the activities of these enzymes fluctuate, cancer progression changes accordingly, affecting treatment (Snider et al., 2019). Scientists have discovered that sphingolipids play an important role in many diseases including kidney disease (Abou Daher et al., 2017), diabetes (Boon et al., 2013), and cancer.

Sphingolipids include two central biologically active lipids that have opposite effects on regulating the death and survival of cancer cells, ceramide (Cer) and sphingosine 1-phosphate (S1P). In 1993, ceramide-induced apoptosis was confirmed in leukemia cells for the first time (Obeid et al., 1993). Subsequent studies showed that ceramides could promote cell death in many different ways, such as by inducing apoptosis, inducing necroptosis, inducing autophagy, causing endoplasmic reticulum stress, and causing cell cycle arrest (Ogretmen, 2018). Two enzymes of the diacylglycerol kinase family, SPHK1 and SPHK2, mediate the production of S1P from ceramide. Endogenous S1P regulates cancer cell signal transduction through S1P receptor (S1PR)-dependent and non-S1PR-dependent pathways, and mediates cancer growth and metastasis. SPHK1 and SPHK2 have opposing effects on the regulation of cell survival. SPHK1 is involved in anti-apoptosis and promotion of angiogenesis (Wang et al., 2020), whereas SPHK2 has pro-apoptotic effects (Liu et al., 2003).

The mechanism by which the sphingolipid pathway regulates cancer is more complicated. The subcellular localization and downstream targets of sphingolipids, especially ceramide and S1P, determine their unique anti-cancer and cancer-promoting functions. The specific functions also depend on the environment and cell type. For instance, mitochondrial accumulation of ceramide promotes cancer cell death by inducing autophagy. The S1P protein produced by SPHK2 binds to receptors on the cell membrane to achieve allosteric-mimicking protein phosphorylation, which increases the stability and function of telomerase, thereby producing a protective effect in cancer cells (Panneer Selvam et al., 2015). In our research, we found that most of the sphingolipid pathway genes in KIRC exist as protective genes, in contrast to their roles in other types of cancers. We also observed that three genes, PLPP1, KDSR, and GBA, are protective factors for KIRC, but their expression levels are upregulated, which appears contradictory. Possible reasons for this observation include: 1) The tumor is heterogeneous, and the expression of the same gene is different in different types of tumors; 2) These protective genes are not tumor suppressors and cannot directly inhibit tumor growth, because these protective genes may function by regulating other genes; and 3) We studied the mRNA expression of related genes, but it is the proteins encoded by these genes that ultimately play a role. Many factors, such as post-translational modifications, epigenetics, and negative-feedback mechanisms, may lead to inconsistent mRNA and protein expression levels. Consequently, we decided to study the role of sphingolipid pathway genes in KIRC further.

DNA methylation is a common form of epigenetic modification. Changes in DNA methylation can affect clinicopathological characteristics and have an impact on patient survival. Also, increased or decreased methylation of two different genes could lead to the same outcome, depending on the particular genes. A study in August 2020 showed that a low methylation level of LAG3 was associated with a lower OS in KIRC (Klumper et al., 2020). The results of another study in September 2020 showed that enhanced Hugl-2 DNA methylation could reduce related mRNA expression and protein content, ultimately promoting the progression of KIRC and reducing the OS of patients with KIRC (Miao et al., 2020). Our research showed that sphingolipid gene methylation is widespread in cancer. Sphingolipid gene methylation affects the expression of cancer mRNA and ultimately has a positive effect on the survival of patients with KIRC. Our research only presents the ultimate result and therefore significantly more work is still required to investigate the entire process, from the beginning of DNA transcription to the final function of the protein. In future research, sphingolipid-related genes should be further studied and verified. Our results may provide new ideas for other related gene methylation and cancer research studies.

In this study, sphingolipid genes were scored based on their mRNA expression levels. KIRC samples were divided into three clusters based on their sphingolipid scores to facilitate subsequent experiments. The survival curves of the three clusters showed that the survival rate associated with inactive clusters was significantly lower than that of active clusters, confirming our previous finding that sphingolipid genes had a protective effect in KIRC. A previous report in April 2020 showed that the MBOAT7 gene could be restricted to reduce the content of arachidonic acid-containing phosphatidylinositol pools in KIRC by altering the lipid metabolism of tumor cells, thereby producing a therapeutic effect (Neumann et al., 2020). However, different opinions exist regarding the effects of renal lipid metabolism on tumor cells. In our study, the survival rate associated with inactive clusters of sphingolipid pathway genes was decreased, appearing to contradict research results showing that HIF2α regulates lipid metabolism, increases lipid storage to maintain endoplasmic reticulum homeostasis, and promotes tumor cell survival (Qiu et al., 2015). This observation may be related to the involvement of sphingolipid-related genes in regulating lipid metabolism of cell membranes, and determining which regulation method has a greater impact on tumor cells will require future research.

The progression of KIRC is closely associated with VHL gene mutations (Nyhan et al., 2008). Studying the levels of transcription factors enables the understanding of pathogenesis more deeply. At present, based on research on transcription factors, some progress in the molecular classification of tumors has occurred (Cancer Genome Atlas Research Network, 2013; Robertson et al., 2017). However, owing to the complexity of cancer genomes, there is no single-molecule method that can pinpoint or describe the driving mechanism of carcinogenesis. Therefore, huge prospects for transcription factor-related aspects worthy of further study exist. Our research shows that there is a correlation between the activity of sphingolipid-related genes and the activity of transcription factors; however, the specific connection between the two requires further research to be accurately described.

Monoclonal antibodies against S1P have been developed, and a recent study showed that targeted therapy delayed the growth of RCC tumors, reduced tumor blood flow, and slowed the growth of sunitinib-resistant tumors (Zhang et al., 2015). We studied the differences in tumor sensitivity to some universally applied targeted drugs used in the treatment of KIRC in three sphingolipid gene clusters associated with different activities. Our results showed that the three clusters exhibited appreciably different drug sensitivities, indicating that patients could be provided with more personalized treatment plans based on their sphingolipid-related gene expression patterns. According to our results, sunitinib may be more effective for patients with inactive sphingolipid genes, whereas axitinib may be more beneficial for patients with active sphingolipid genes. Tumors are often accompanied by the infiltration of surrounding inflammatory cells. Initially, scientists thought that these immune cells helped the body resist tumors. Later, it was found that most tumors are not regarded as foreign bodies by the immune system, and that inflammatory immune cell infiltration promotes tumor growth and metastasis. Current studies have confirmed the role of sphingolipid family members in specific inflammatory processes including: 1) participating in the migration of immune cells, 2) aiding in the identification of exogenous factors, and 3) participating in the activation/differentiation of immune cells (Grosch et al., 2018). By observing the growth of CMS4-met-derived soft tissue sarcoma tumors in a mouse model, ceramide was found to inhibit the function of myeloid-derived suppressor cells, leading to the weakening of autophagy and the induction of endoplasmic reticulum stress, thereby enhancing the function of cytotoxic T lymphocytes and producing an antitumor effect (Liu et al., 2016). In this study, we explored the correlation between factors related to immune cell infiltration and sphingolipid-related genes. We analyzed the correlation between the sphingolipid pathway score and immune cell infiltration and found that the sphingolipid correlation score was positively correlated with the infiltration of type II IFN-responsive cells and mast cells, and negatively correlated with HLA and CD8 cells. Interferon-γ (IFN-γ) has been widely studied for its role in regulating immune status and antitumor immunity. Mast cells can mediate tumor growth via the immune pathway. Downregulation of HLA genes may lead to reduced antigen presentation, thereby promoting immune evasion and ultimately resulting in a series of undesirable consequences, including cancer-promoting effects (Campoli and Ferrone, 2008; Mehta et al., 2008). CD8 cells play a critical role in the antitumor immune response as they can directly kill tumor cells, and immunotherapy for metastatic KIRC is still used as the first-line treatment at present. Currently, targeted therapy for the sphingolipid pathway has a significant effect on delaying tumor growth (Visentin et al., 2006; Venant et al., 2015), and has received increasing attention. Our research provides new avenues for KIRC immunotherapy through analysis of these immune-related factors.

Abnormal expression of HDAC is closely associated with cancer (Barneda-Zahonero and Parra, 2012). HDAC inhibitors can improve the ability of immune cells to recognize tumors, which may indirectly promote antitumor activity (Zhao and Zhang, 2019). The results of our research indicated that most oncogenes, tumor suppressor genes, and HDACs were related to the sphingolipid pathway. Therefore, HDAC inhibitors could be used to treat tumors specifically, and our research results could provide a reference for future treatments. For example, the expression level of HDAC10 in the sphingolipid gene-inactive group was significantly higher than that in the sphingolipid gene-active group, suggesting that the use of HDAC10 inhibitors may be more helpful in the former group of patients.

We used LASSO Cox regression analysis to build a model that could predict the survival rate of patients with KIRC, and the area under the ROC curves derived from the model indicated that it had high predictive value. Finally, we included RS, patient age, tumor grade, and stage into a nomogram to predict the 5-, 7-, and 10-year survival rates of patients with KIRC. Currently, KIRC has other risk and survival predictors based on different mechanisms (Xu et al., 2020; Wu et al., 2021). For example, constructed an immune prognosis prediction model based on 14 immune-related groups, and proved that the model could be effectively and efficiently used to predict the survival outcome and immunotherapy response of patients with KIRC Feng et al. (2021). Overall, our sphingolipid prognostic features have a higher predictive accuracy for patients with KIRC than the abovementioned prognostic features.

Our study had some limitations. First, the research was purely bioinformatic, and the scientific hypothesis was not confirmed by biological experiments. Second, the sample size of the sequencing data was limited. Third, the lack of further studies on biological samples of metastatic sites, such as lungs, bones, and brain, make this research incomplete. Finally, the limitations of a single omics analysis are also inherent limitations of this study.

In conclusion, our research found that sphingolipid genes are mostly protective genes in KIRC. Sphingolipid gene methylation has a positive effect on the prognosis of KIRC, and the activity of sphingolipid genes and transcription factors, drug sensitivity, immune cell infiltration, classic cancer genes, and histone modifications are closely related. Our current model enriches existing prognostic models and may provide more comprehensive and useful recommendations for the development of personalized treatments for patients with KIRC.

## Data Availability

The datasets presented in this study can be found in online repositories. The names of the repository/repositories and accession number(s) can be found in the article/Supplementary Material.

## Glossary

**KIRC**: Kidney renal clear cell carcinoma
**CNV**: Copy number variation
**SNV**: Single nucleotide variation
**TCGA**: The cancer genome atlas
**LASSO**: Least absolute shrinkage and selection operator
**ROC**: Receiver operating characteristic
**RS**: Risk score
**AUC**: Area under the curve
**OS**: overall survival
**PFS**: Progression-free survival
**DFS**: Disease-free survival
**HDAC**: Histone deacetylases
**SIRT**: Sirtuin
**GCS**: Glucosylceramide synthase
**Cer**: Ceramide
**S1P**: Sphingosine 1-phosphate
**TME**: Tumor microenvironment
**SPHK1**: Sphingosine Kinase 1
**CERS5**: Ceramide synthase 5
**PLPP1**: Phospholipid phosphatase 1
**SGMS1**: Sphingomyelin synthase 1
**SGMS2**: Sphingomyelin synthase 2
**SERINC1**: Serine incorporator 1
**KDSR**: 3-Ketodihydrosphingosine reductase
**ccRCC**: clear cell renal cell carcinoma
**GSEA**: Gene set enrichment analysis
**BRCA**: Breast cancer
**PRAD**: Prostate adenocarcinoma
**LUAD**: Lung adenocarcinoma
**DFI**: Disease-free interval
**DSS**: Disease special survival
**GDSC**: Genomics of drug sensitivity in cancer
**CTRP**: Cancer therapeutics response portal
**IFN**: Interferon
**DLBC**: Lymphoid neoplasm diffuse large B-cell lymphoma
**SKCM**: Skin cutaneous melanoma
**UCEC**: Uterine corpus endometrial carcinoma
**THCA**: Thyroid carcinoma
**HR**: Hazard ratio
**GBA**: Glucosylceramidase beta
**GAL3ST1**: Galactose-3-O-sulfotransferase 1
**SPTLC3**: Serine palmitoyltransferase long chain base subunit 3
**SPTLC2**: Serine palmitoyltransferase long chain base subunit 2
**UGCG**: UDP-Glucose ceramide glucosyltransferase
**CERS6**: Ceramide synthase six
**ASAH1**: N-acylsphingosine amidohydrolase 1
**UGT8**: UDP glycosyltransferase eight
**SPTLC1**: Serine palmitoyltransferase long chain base subunit 1
**SGPP2**: Sphingosine-1-phosphate phosphatase 2
**PPP2CA**: Protein phosphatase 2 catalytic subunit alpha
**ERBB3**: Erb-B2 receptor tyrosine kinase 3
**FGFR3**: Fibroblast growth factor receptor 3
**PPARG**: Peroxisome proliferator activated receptor gamma
**ESR1**: Estrogen receptor 1
**STAT3**: Signal transducer and activator of transcription 3: Signal transducer and activator of transcription 3
**AR**: Androgen receptor
**RARB**: Retinoic acid receptor beta
**EGFR**: Epidermal growth factor receptor
**KLF4**: Kruppel like factor 4
**RXRA**: Retinoid X receptor alpha
**FOXM1**: Forkhead box M1
RARA: Retinoic acid receptor alpha
**FGFR1**: Fibroblast growth factor receptor 1
**RARG**: Retinoic acid receptor gamma
**TP63**: Tumor protein P63
**RXRB**: Retinoid X receptor beta
**FOXA1**: Forkhead box A1
**GATA6**: GATA binding protein six
**ESR2**: Estrogen receptor 2
**GATA3**: GATA binding protein 3
**ERBB2**: Erb-B2 receptor tyrosine kinase 2
**HIF1A**: Hypoxia inducible factor 1 subunit alpha
**PGR**: Progesterone receptor
**CCND1**: Cyclin D1
**BRAF**: B-raf proto-oncogene, serine/Threonine kinase
**AKT1**: AKT serine/Threonine kinase 1
**MYC**: MYC proto-oncogene, BHLH transcription factor
**KRAS**: KRAS proto-oncogene, GTPase
**MTOR**: Mechanistic target of rapamycin kinase
**PIK3CA**: Phosphatidylinositol-4,5-bisphosphate 3-kinase catalytic subunit alpha
**VEGFA**: Vascular endothelial growth factor A
**PTEN**: Phosphatase and tensin homolog
**VHL**: Von hippel-lindau tumor suppressor
**CTNNB1**: Catenin beta 1
**HRAS**: HRas proto-oncogene, GTPase
**STAT3**: Signal transducer and activator of transcription 3: Signal transducer and activator of transcription 3
**TP53**: Tumor protein P53
